# Can Adverse Effects of Acidity and Aluminum Toxicity Be Alleviated by Appropriate Rootstock Selection in Cucumber?

**DOI:** 10.3389/fpls.2016.01283

**Published:** 2016-08-29

**Authors:** Youssef Rouphael, Elvira Rea, Mariateresa Cardarelli, Michael Bitterlich, Dietmar Schwarz, Giuseppe Colla

**Affiliations:** ^1^Department of Agricultural Sciences, University of Naples Federico IINaples, Italy; ^2^Centro di Ricerca per lo Studio delle Relazioni tra Pianta e Suolo, Consiglio per la Ricerca in Agricoltura e l'Analisi dell'Economia AgrariaRome, Italy; ^3^Leibniz Institute of Vegetable and Ornamental CropsGroßbeeren, Germany; ^4^Department of Agricultural and Forestry Sciences, University of TusciaViterbo, Italy

**Keywords:** Al tolerance, chlorophyll index, *Cucumis sativus* L., graft combination, membrane stability, mineral composition, pH level

## Abstract

Low-pH and aluminum (Al) stresses are the major constraints that limit crop yield in acidic soils. Grafting vegetable elite cultivars onto appropriate rootstocks may represent an effective tool to improve crop tolerance to acidity and Al toxicity. Two greenhouse hydroponic experiments were performed to evaluate growth, yield, biomass production, chlorophyll index, electrolyte leakage, mineral composition, and assimilate partitioning in plant tissues of cucumber plants (*Cucumis sativus* L. “Ekron”) either non-grafted or grafted onto “P360” (*Cucurbita maxima* Duchesne × *Cucurbita moschata* Duchesne; E/C) or figleaf gourd (*Cucurbita ficifolia* Bouché; E/F). Cucumber plants were cultured in pots and supplied with nutrient solutions having different pH and Al concentrations: pH 6, pH 3.5, pH 3.5 + 1.5 mM Al, and pH 3.5 + 3 mM Al (Experiment 1, 14 days) and pH 6, pH 3.5, and pH 3.5 + 0.75 mM Al (Experiment 2, 67 days). Significant depression in shoot and root biomass was observed in response to acidity and Al concentrations, with Al-stress being more phytotoxic than low pH treatment. Significant decrease in yield, shoot, and root biomass, leaf area, SPAD index, N, K, Ca, Mg, Mn, and B concentration in aerial parts (leaves and stems) in response to low pH with more detrimental effects at pH 3.5 + Al. Grafted E/C plants grown under low pH and Al had higher yield, shoot, and root biomass compared to E/F and non-grafted plants. This better crop performance of E/C plants in response to Al stress was related to (i) a reduced translocation of Al from roots to the shoot, (ii) a better shoot and root nutritional status in K, Ca, Mg, Mn, and Zn concentration, (iii) a higher chlorophyll synthesis, as well as (iv) the ability to maintain cell membrane stability and integrity (lower electrolyte leakage). Data provide insight into the role of grafting on Al stress tolerance in cucumber.

## Introduction

Acidic soils represent 50% of the earth's arable land (Inostroza-Blancheteau et al., 2012), and in those soils aluminum (Al) toxicity is the main factor restricting crop productivity (Kochian, 1995). In acidic soils with pH lower than 5, Al-containing minerals (e.g., aluminosilicates) are solubilized in the phytotoxic form Al^3+^ (Kochian, 1995; Seguel et al., 2013). The root is the first organ feeling Al^3+^ toxicity, thus conditioning stress sensitivity and hampering crop productivity (Delhaize and Ryan, 1995; Rengel et al., 2015). For many plant species, Al concentration in the range of 1–2 mg L^−1^ can inhibit root elongation by damaging the cell structure of the root apex and thus influencing water and nutrient uptake (Kochian et al., 2015; Rengel et al., 2015). Al also interferes with cell membrane stability, enzyme function, and the synthesis of chlorophyll (Simon et al., 1994; Rouphael et al., 2015). Several authors reported that exposure to Al^3+^ in the rooting medium strongly inhibit Ca^2+^ and Mg^2+^ fluxes across the plasma membrane of root cells (Rengel and Elliott, 1992; Horst et al., 2010; Bose et al., 2011). Similarly, Al toxicity can decease or even block the uptake of K^+^, since it interacts with various different plasma-membrane channel proteins (Kochian et al., 2005). This may decrease cations uptake leading to nutrient deficiencies, thus affecting metabolism and productivity of crops. However, plant species have different degrees of adaptation to Al; these differences are often related to the specific mechanisms developed for mitigating stress (Panda and Matsumoto, 2007). The mechanisms conferring tolerance to Al have been classified into mechanisms of *exclusion* and those of *intracellular tolerance* (Kochian, 1995). Exclusion mechanisms not allowing Al entering the symplast can take place by exudation of Al-chelating compounds (e.g., organic acids), raising the pH in the rhizospheric environment and by binding of Al in the cell wall itself (Inostroza-Blancheteau et al., 2012; Brunner and Sperisen, 2013; Kochian et al., 2015). Internal tolerance mechanisms include those that bind the Al entering the root cells as well as the sequestration of Al in subcellular compartments (e.g., vacuoles).

To overcome the limitations of Al toxicity several solutions have been proposed. Amendments such as gypsum, lime, and phosphate fertilizers are common agricultural practices used to raise the soil pH causing the Al to become insoluble (Nawrot et al., 2001). Liming is only efficient in the topsoil, while it does not remedy the subsoil acidity (>1 m), since deep incorporation of lime is difficult to realize and very costly (Choudhary and Singh, 2011; Yang et al., 2013). Another possible solution to alleviate the negative effects of acid soils would be the use Al-tolerant cultivars obtained by breeding and/or biotechnological approaches (Choudhary and Singh, 2011). However, the long time needed for the breeding of Al-tolerant cultivars as well as the complexity of the Al-trait make this task extremely difficult. In order to overcome this situation more rapidly, grafting elite vegetable cultivars onto rootstocks tolerating higher Al concentrations and low pH could be a key tool in alleviating the effects of acidity and Al toxicity on crop productivity.

By selecting suitable rootstocks, grafting can control scion morphology (Albacete et al., 2015), increase productivity and fruit quality (Colla et al., 2008; Proietti et al., 2008; Kyriacou et al., 2016), improve nitrogen use efficiency (Colla et al., 2010a, 2011), and also induce tolerance against several abiotic stresses, among them nutrient toxicity, heavy metals, and alkalinity (Edelstein et al., 2005, 2007; Rouphael et al., 2008a,b; Colla et al., 2010b; Savvas et al., 2010, 2013; Kumar et al., 2015a). Therefore, we hypothesized that the effectiveness of root genotypes to enhance the uptake of nutrients and to limit the Al accumulation in aerial parts may be improved by grafting onto suitable rootstocks.

To verify the above hypothesis, two hydroponic greenhouse experiments were conducted (1) to assess the effects of grafting combinations on morphological traits of cucumber at early development stage in response to different nutrient solution pH and Al concentrations; and (2) to elucidate the agronomical, physiological, and mineral composition changes of cucumber mediated by grafting under acidity and Al toxicity conditions using long-term treatments.

## Materials and methods

### Plant material selection and growth conditions

A short- and a long-term experiment were performed to characterize the response to acidity and Al toxicity in cucumber (*Cucumis sativus* L.) either non-grafted or grafted onto vigorous rootstocks. Both experiments were conducted in a polyethylene greenhouse at the experimental station of the University of Tuscia, located in Viterbo (latitude 42°25′N, longitude 12°08′E, altitude 310 m). Inside the greenhouse the mean air temperatures amounted to 25°C varying between 18 and 30°C, while the mean day/night relative humiditiy was 55/80%.

In the short-term experiment (Experiment 1), *C. sativus* L. “Ekron” (E; Enza Zaden, Verona, Italy) was grafted onto the commercial rootstock “P360” (C; *Cucurbita maxima* × *Cucurbita moschata*; Società Agricola Italiana Sementi, Cesena, Italy), whereas in the long-term experiment (Experiment 2) cucumber “Ekron” was grafted onto the “P360” and also onto the figleaf gourd (F) (*Cucurbita ficifolia* Bouché; Società Agricola Italiana Sementi, Cesena, Italy) using the procedure of insertion grafting (Lee et al., 2010). In both experiments the non-grafted “Ekron” was used as control. The *Cucurbita* hybrid “P360” and the figleaf gourd were selected as the most representative commercial rootstocks used in the Mediterranean basin and Asia, respectively (Lee et al., 2010). In Experiment 1, grafted and non-grafted seedlings were transplanted 20 days after sowing, at the two true-leaf stages into plastic pots of 2 L volume (one plant per pot) filled with quartziferous sand and placed over 16 cm wide and 5 m long benches at a plant density of 11 plants m^−2^. In Experiment 2, cucumber seedlings were transplanted on 22 May into pots containing 17.7 L of quartziferous sand. Pots were disposed in double rows at a plant density of 2.5 plants m^−2^, as used commercially for cucumber under greenhouse conditions. Cucumber plants were grown as vertical cordons and trained to the *umbrella system* as described by Rouphael et al. (2010).

### Experimental design, treatments, and nutrient solution management

Experiment 1 contained eight treatments, derived by the factorial combination of two grafting combinations (grafted E/C and non-grafted E plants) and four nutrient solutions (pH 6.0, pH 3.5, pH 3.5 + 1.5 mM Al, and pH 3.5 + 3.0 mM Al). Any treatment had four replications, amounting to a total of 32 experimental unit plots with eight plants each (*n* = 256 plants). In Experiment 2, nine treatments, derived by the factorial combination of three grafting combinations (non-grafted E, grafted E/C and E/F) and three nutrient solutions (pH 6.0, pH 3.5, and pH 3.5 + 0.75 mM Al) were compared with four replications, amounting to a total of 36 experimental unit plots (*n* = 180 plants). Each experimental unit consisted of five plants.

Plants were drip-irrigated automatically 2-4 times per day in Experiment 1 and 3–10 times in Experiment 2, to ensure adequate substrate moisture (Colla et al., 2012, 2013). The basic nutrient solution (pH 6.0) was a modified Hoagland and Arnon formulation containing the following macro- and micro-nutrients: 13.0 mM N–${\text{NO}}_{3}^{-}$, 1.6 mM S, 0.3 mM P, 4.3 mM K, 4.0 mM Ca, 1.3 mM Mg, 20 μM Fe, 9 μM Mn, 0.3 μM Cu, 1.6 μM Zn, 20 μM B, and 0.3 μM Mo. The low pH (3.5) treatments had the same basic nutrient composition plus HCl which was added to decrease the nutrient solution pH, thus simulating the effects of acidity. The Al treatments were generated by adding AlCl_3_·6 H_2_O to the basic nutrient solution. The stressed treatments (pH 3.5 and pH 3.5 + Al) started at transplanting in Experiment 1, and 7 days after transplanting in Experiment 2.

### Yield assessment and growth measurements

In Experiment 2, fruits of all plants were harvested manually 2–3 times per week from 16 June to 25 July. The number of fruits per plant, the mean fruit weight, and the marketable yield were recorded. At final harvest, all plants were separated into leaves, stems and roots, and dried in a forced-air oven at 80°C for 72 h for biomass determination. Root-to-shoot ratio was calculated by dividing root dry weight by the sum of leaf and stem dry weights. The final leaf area was also measured with an electronic leaf area meter (Delta-T Devices Ltd., Cambridge, UK).

### Fruit quality analysis

On 1 July (43 days after transplanting), five fruits were sampled from each plot for quality analysis. Fruit shape index (SI) was defined by the ratio of equatorial and longitudinal lengths. Fruit firmness was measured using a penetrometer (Bertuzzi FT 011; Brugherio, Milan, Italy), fitted with an 8 mm-diameter round-head probe. A part of the homogenate prepared under low speed and filtered under cheesecloth was used for determining the total soluble solid content at 20°C using an Atago N1 refractometer (Atago Co. Ltd., Japan). The pH of the juice was measured with a pH electrode (HI-9023, Hanna Instruments, Padova, Italy). The titratable acidity of the juice, expressed as % w/v malic acid content, was determined by titration of an aliquot of 25 mL with 0.1 M NaOH to a pH endpoint of 8.1 on an automatic titrator. Fruit dry matter percentage was also determined by drying a part of the homogenate in a forced air oven at 8°C for 72 h.

### Analysis of aluminum and mineral nutrient concentrations in plant tissues

The dried plant tissues: leaf, stem, fruit, and root tissues were ground in a Wiley mill to pass through a 20-mesh screen, then 0.5 g samples were analyzed for the following macro- micro-nutrients and toxic element: N, P, K, Ca, Mg, Fe, Mn, Zn, B, and Al. Nitrogen (total N) concentration in the four plant tissues was determined after mineralization with sulfuric acid (H_2_SO_4_, 96%, Carlo Erba Reagents, Cornaredo, Milan, Italy) in the presence of potassium sulfate (K_2_SO_4_) and a low concentration of copper (Cu) according to the Kjeldahl method (Bremner, 1965). Phosphorus, K, Ca, Mg, Fe, Mn, Zn, B, and Al were determined by dry ashing at 400°C for 24 h, dissolving the ash in HNO_3_ (1:20 w/v) and assaying the solution obtained using an inductively coupled plasma emission spectrophotometer (ICP Iris, Thermo Optek, Milan, Italy; Karla, 1998).

### SPAD index measurement

One day before the end of Experiment 2 the Soil Plant Analysis Development (SPAD index), a non-destructive and indirect measurement of leaf chlorophyll content, was measured on the fully expanded leaves by means of a portable chlorophyll meter SPAD-502 (Konica-Minolta corporation, Ltd., Osaka, Japan). Measurements were made at the central point of the leaflet between the midrib and the leaf margin. Fifteen leaves were randomly measured and averaged to a single SPAD value for each treatment.

### Determination of electrolyte leakage

At the same date of the SPAD index measurement, the membrane integrity in leaves was measured in terms of electrolyte leakage as described by Lutts et al. (1995). Briefly, 10 pieces of equal-sized leaves (10 × 10 mm) collected from four plants per plot were placed in individual vials containing 10 ml of distilled water. The vials were incubated at room temperature (25°C) for 24 h with continuously shaking and the initial electrical conductivity (EC_1_) of the bathing solution was measured using a conductivity meter (HI991301; Hanna Instruments, Padova, Italy). To measure the total electrolytes released from leaf tissues, vials were then autoclaved at 120°C for 20 min and cooled at 25°C to obtain the final electrical conductivity (EC_2_). The EL was calculated using the following formula: EL (%) = (EC_1_/EC_2_) × 100.

### Statistical analysis

All experimental data were subjected to a two-way ANOVA using the SPSS software package (SPSS, 2001). When ANOVA indicated that either nutrient solution or graft combination or their interaction was significant, mean separation was performed using the Duncan's multiple range test at *p* = 0.05 on each of the significant variables measured.

## Results

### Growth response and leaf symptoms

**Figure 1:**
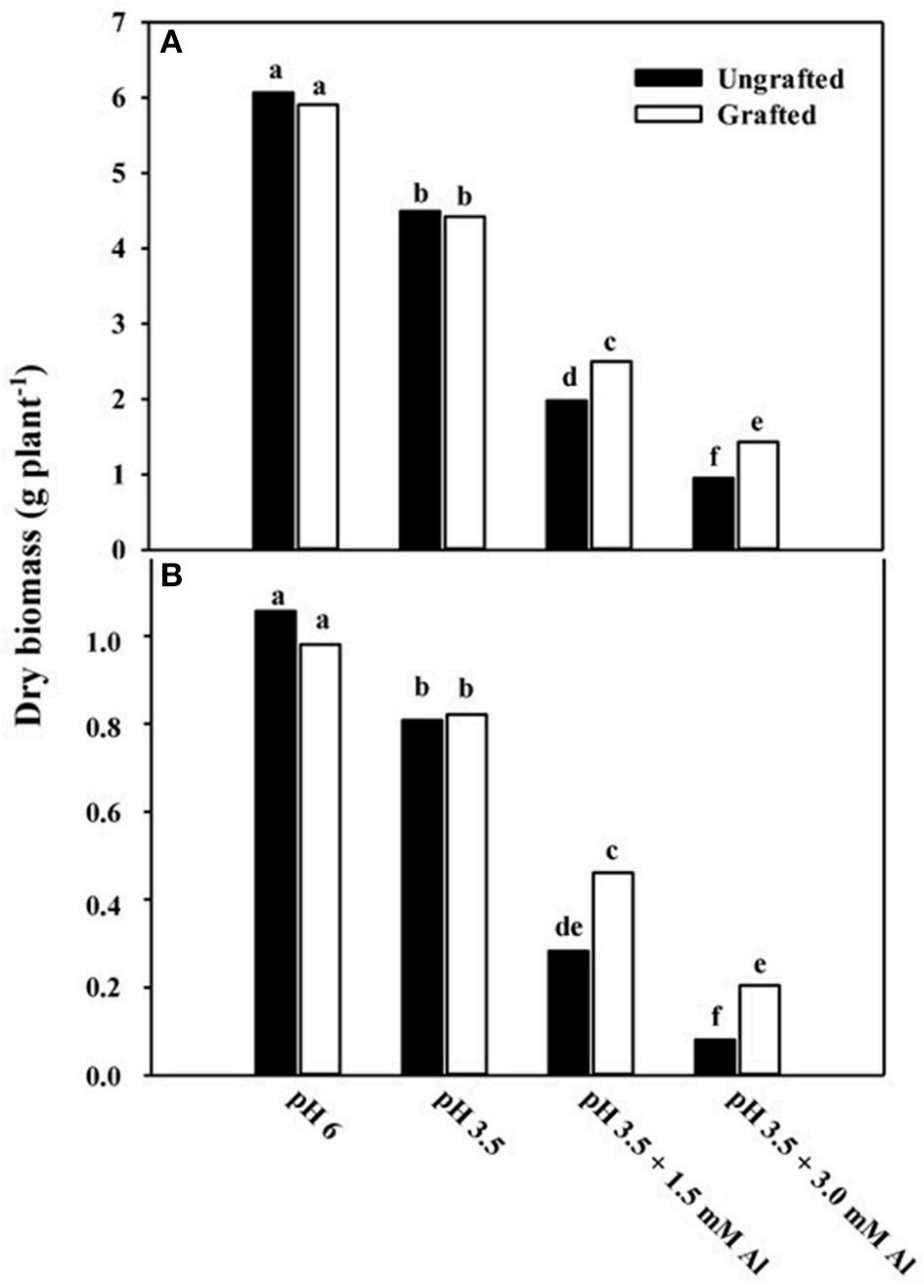
**Effects of grafting combination, nutrient solution pH and aluminum concentration on shoot (A) and root (B) dry mass of cucumber plants grown in experiment 1 (14 days after transplanting)**. Different letters indicate significant differences according to Duncan's test (*P* = 0.05). Values are the means of four replicate samples.

**Figure 2:**
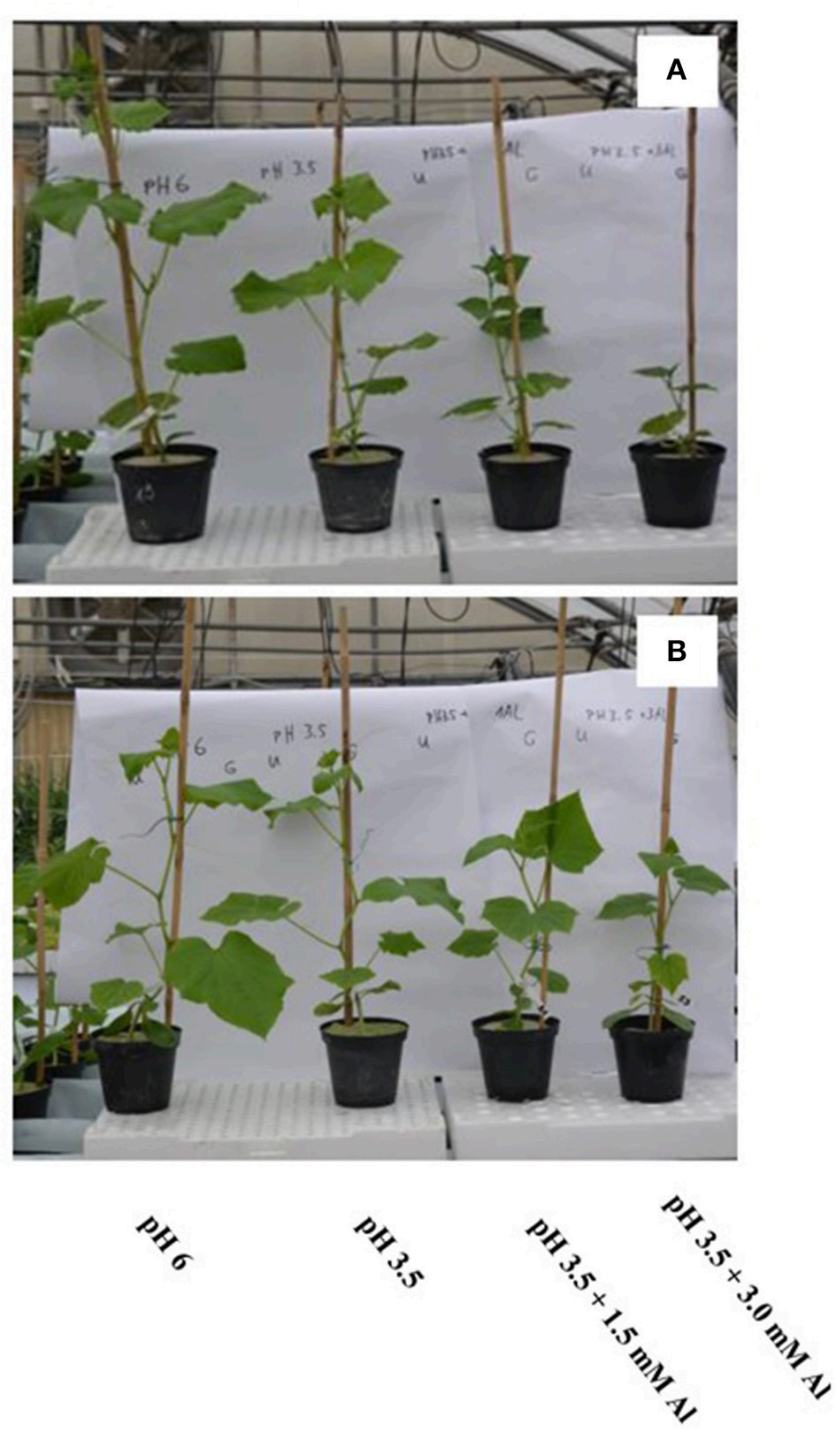
**Effects of nutrient solution pH and aluminum concentration on non-grafted (A) and grafted (B) cucumber plants in experiment 1 (14 days after transplanting)**.

**Figure 3:**
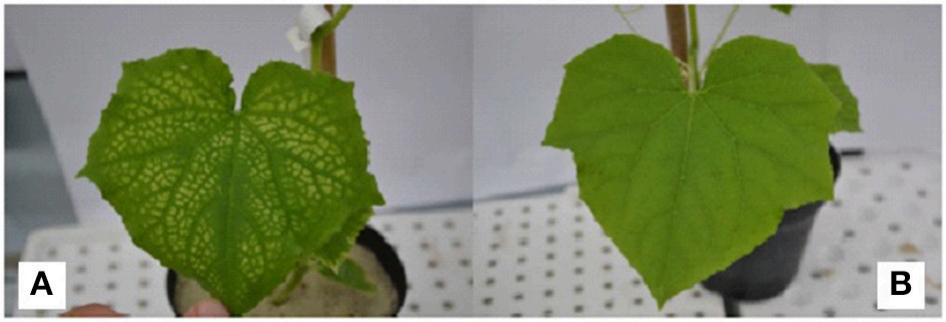
**Fully expanded leaves of non-grafted (A) and grafted (B) cucumber plants grown in experiment 1 (14 days after transplanting) and treated with a nutrient solution at pH 3.5**.

In Experiment 1, shoot and root dry mass of cucumber plants were significantly (*p* < 0.01) affected by nutrient solution and grafting combination interaction (data not shown). Shoot dry mass at pH 3.5 in non-grafted and grafted plants was decreased by 26 and 25% over the pH 6.0 treatment (Figure 1A). Moreover, shoot dry mass was reduced by 67 and 58% over the pH 6.0 treatment at pH 3.5 + 1.5 mM Al in non-grafted and rootstock-grafted plants, and further decreased by 84 and 76% at pH 3.5 + 3.0 mM Al (Figure 1A). Similarly, root dry mass declined at pH 3.5 + 1.5 mM Al and especially at pH 3.5 + 3.0 mM Al, however, less in rootstock-grafted plants (Figure 1B). Plant growth traits in particular plant height, leaf area and number were significantly (*p* < 0.01) affected by nutrient solution and grafting combination interaction (data not shown). The biometric plant traits decreased under acidity and Al concentration especially at pH 3.5 + 3.0 mM Al (data not shown), however, the reduction in plant growth was less pronounced in rootstock-grafted plants (Figure 2). The most evident symptom of adverse low pH conditions (i.e., acidity) is a yellowing between leaf veins giving the leaves a “*marbled appearance*,” indicating magnesium deficiency. These symptoms recorded after 14 days in plants grafted onto pumpkin rootstock were less pronounced than those observed in the non-grafted plants (Figure 3).

In Experiment 2, shoot and root dry mass at the end of the growing season were significantly affected by nutrient solution, grafting combination, and their interactions, whereas the root-to-shoot ratio (R/S) was significantly influenced by both main factors (Table 1). For instance, the shoot dry mass was decreased by 30, 25, and 20% over control at low adverse pH (3.5) treatment in non-grafted E, E/F, and E/C, and strongly decreased by 77, 73, and 66% at pH 3.5 + Al (Table 1). Consistent with the shoot dry mass, the root dry weight of cucumber plants decreased by 16, 11, and 8% at pH 3.5 in E, E/F, and E/C, respectively and decreased substantially by 65, 48, and 36% at pH 3.5 + Al (Table 1). The highest R/S ratio was recorded at pH 3.5 + Al compared to pH 6.0 and pH 3.5 treatments, whereas among grafting combination the E/C combination exhibited the highest R/S ratios (Table 1).

**Table 1:** **Effects of grafting combination, nutrient solution pH, and aluminum concentration on shoot and root biomass dry weight, and on the root-to-shoot ratio (R/S) of cucumber plants grown in experiment 2**.

**Solution**	**Graft combination**	**Shoot (g plant^−1^)**	**Root (g plant^−1^)**	**R/S**
pH 6.0	E	101.1a	6.3a	0.06
	E/F	96.0a	5.6ab	0.06
	E/C	97.8a	6.4a	0.07
	Mean	98.3a	6.1a	0.06b
pH 3.5	E	70.7c (30)	5.3b	0.07
	E/F	72.3c (25)	5.0bc	0.07
	E/C	77.8b (20)	5.9ab	0.08
	Mean	73.6b	5.4b	0.07b
pH 3.5 + Al	E	23.2e (77)	2.2d	0.09
	E/F	24.7e (73)	2.9d	0.12
	E/C	33.6d (66)	4.1c	0.12
	Mean	27.2c	3.1c	0.11a
**SIGNIFICANCE**				
Solution (S)		^***^	^***^	^***^
Graft combination (G)		^**^	^*^	^*^
S × G		^*^	^**^	ns

*E, cucumber hybrid “Ekron” (Cucumis sativus L.); E/F, “Ekron” grafted onto figleaf gourd rootstock (Cucurbita ficifolia Bouché); E/C, “Ekron” grafted onto squash hybrid rootstock “P360” (Cucurbita maxima Duch. × Cucurbita moschata Duch.)*.

*Values are the means of four replicate samples. The percentage of reduction in solution treatments having pH 3.5 and pH 3.5 + Al with respect to the solution treatment having pH 6.0 (control) are reported in parenthesis*.

*Means within columns separated using Duncan's multiple range test, P = 0.05*.

*ns*.,

*, **, *** *Nonsignificant or significant at P < 0.05, 0.01, or 0.001, respectively*.

### Marketable yield and yield components

In grafted and non-grafted plants, the marketable yield diminished in response to a decrease of the nutrient solution pH with more adverse effects at pH 3.5 + Al (Table 2). The reduced marketable yield was mostly caused by a reduced number of fruits per plant and mean weight (Table 2). Moreover, at pH 3.5 and pH 3.5 + Al, the percentage of yield reduction in comparison to the pH 6.0 treatment was lower in E/C (25 and 71%, respectively) than in E/F (27 and 79%, respectively) and non-grafted plants (32 and 81%, respectively; Table 2). Under Al toxicity conditions, plants of E/C had the highest marketable fruit yield (+50%) compared to those at E and E/F combinations (Table 2). The relatively low yield of non-grafted and E/F plants at pH 3.5 + Al was attributed to a reduction in the fruit mean weight and not to a change in the number of fruit per plant (Table 2).

**Table 2:** **Effects of grafting combination, nutrient solution pH, and aluminum concentration on marketable fruit yield, marketable fruit mean weight, and number of cucumber plants grown in experiment 2**.

**Solution**	**Graft combination**	**Yield (kg plant^−1^)**	**Fruit**	
			**Number (n. plant^−1^)**	**Mean weight (g fruit^−1^)**
pH 6.0	E	4.04a	12.5a	323.2
	E/F	3.91a	12.4a	315.3
	E/C	3.90a	12.1a	322.3
	Mean	3.95a	12.3a	320.3a
pH 3.5	E	2.73c (32)	8.6b	317.4
	E/F	2.85bc (27)	9.0b	316.7
	E/C	2.94b (25)	8.5b	345.9
	Mean	2.84b	8.7b	326.7a
pH 3.5 + Al	E	0.77e (81)	3.0d	256.7
	E/F	0.80e (79)	3.8c	210.5
	E/C	1.17d (71)	4.1c	285.4
	Mean	0.91c	3.6c	250.9b
**SIGNIFICANCE**				
Solution (S)		^***^	^***^	^***^
Graft combination (G)		ns	ns	^*^
S × G		^**^	^*^	ns

*E, cucumber hybrid “Ekron” (Cucumis sativus L.); E/F, “Ekron” grafted onto figleaf gourd rootstock (Cucurbita ficifolia Bouché); E/C, “Ekron” grafted onto squash hybrid rootstock “P360” (Cucurbita maxima Duch. × Cucurbita moschata Duch.)*.

*Values are the means of four replicate samples. The percentage of reduction in solution treatments having pH 3.5 and pH 3.5 + Al with respect to the solution treatment having pH 6.0 (control) are reported in parenthesis*.

*Means within columns separated using Duncan's multiple range test P = 0.05*.

*ns*.,

*, **, *** *Nonsignificant or significant at P < 0.05, 0.01, or 0.001, respectively*.

### Fruit quality

No significant changes were observed for titratable acidity (Table 3). The fruit SI was only influenced by nutrient solution pH and Al concentration with the highest values observed in cucumbers treated with pH 3.5 + Al compared to pH 3.5 and pH 6.0 treatments (Table 3). Under pH 3.5 and Al toxicity, the fruit firmness, dry matter, and total soluble solid contents decreased, but they were not significantly affected by the grafting onto figleaf gourd or pumpkin (Table 3). Moreover, when averaged over nutrient solution pH and Al concentration the highest fruit dry matter content was recorded with E/F, followed by E/C combination, whereas the lowest values were observed with non-grafted plants.

**Table 3:** **Effects of grafting combination, nutrient solution pH, and aluminum concentration on fruit shape index (SI), firmness, dry matter content (DMC), total soluble solids (TSS) content, juice pH, and titratable acidity (TA) of cucumber fruits grown in experiment 2**.

**Solution**	**Graft combination**	**SI**	**Firmness (N cm^−2^)**	**DMC (%)**	**TSS (°brix)**	**pH**	**TA (%)**
pH 6.0	E	0.24	1.53	4.41	3.87a	6.12	0.11
	E/F	0.23	1.56	4.34	3.63a	6.12	0.09
	E/C	0.23	1.52	4.24	3.70a	6.18	0.09
	Mean	0.23b	1.54a	4.33a	3.74a	6.14a	0.10
pH 3.5	E	0.23	1.32	3.43	2.92d	5.81	0.08
	E/F	0.23	1.37	3.70	3.14c	5.90	0.08
	E/C	0.23	1.60	3.77	3.17c	5.97	0.11
	Mean	0.23b	1.43ab	3.63b	3.08b	5.89b	0.09
pH 3.5 + Al	E	0.27	1.29	3.34	3.22c	5.52	0.09
	E/F	0.28	1.23	4.34	3.53bc	5.28	0.10
	E/C	0.24	1.43	3.78	3.09cd	5.61	0.08
	Mean	0.26a	1.31b	3.82b	3.28b	5.47c	0.09
**SIGNIFICANCE**							
Solution (S)		^***^	^*^	^***^	^***^	^***^	ns
Graft combination (G)		ns	ns	^*^	ns	ns	ns
S × G		ns	ns	ns	^**^	ns	ns

*E, cucumber hybrid “Ekron” (Cucumis sativus L.); E/F, “Ekron” grafted onto figleaf gourd rootstock (Cucurbita ficifolia Bouché); E/C, “Ekron” grafted onto squash hybrid rootstock “P360” (Cucurbita maxima Duch. × Cucurbita moschata Duch.)*.

*Values are the means of four replicate samples*.

*Means within columns separated using Duncan's multiple range test, P = 0.05*.

*ns*.,

*, **, *** *Nonsignificant or significant at P < 0.05, 0.01, or 0.001, respectively*.

### Aluminum uptake and distribution

Al treatment increased Al concentration in leaves, stems, and roots but did not affect it in fruits (Table 4). The partitioning of Al within the plant tissues was in the following order: roots > leaves > stems > fruits. The highest shoot (leaf and stem) and root Al concentrations were observed in cucumber plants treated with pH 3.5 + Al (Table 4). There was a marked influence of grafting combination on the accumulation of Al in aerial vegetative plant parts, whereas no statistical effects were observed in fruits and roots. Particularly, the accumulation of Al in leaf tissue at pH 3.5 + Al, with respect to pH 6.0, was significantly lower in E/C plants (22%), in comparison to E/F and non-grafted plants (72 and 87%, respectively).

**Table 4:** **Effects of grafting combination, nutrient solution pH, and aluminum concentration on Al distribution in leaf, stem, fruit, and root tissues of cucumber plants grown in experiment 2**.

**Solution**	**Graft combination**	**Al (mg kg^−1^ DW)**			
		**Leaves**	**Stems**	**Fruits**	**Roots**
pH 6.0	E	85.7c	10.6	6.2	263.7
	E/F	80.5c	13.7	6.2	271.1
	E/C	87.2c	7.8	6.0	236.0
	Mean	84.5b	10.7b	6.1	257.0b
pH 3.5	E	90.5c	10.1	6.1	303.4
	E/F	88.0c	10.6	7.3	468.7
	E/C	93.0c	9.6	7.5	734.0
	Mean	90.5b	10.1b	6.9	502.1b
pH 3.5 + Al	E	160.0a	22.5	7.7	7697.0
	E/F	138.3b	19.8	10.0	6575.7
	E/C	106.4c	16.6	7.1	5395.8
	Mean	134.9a	19.6a	8.9	6556.1a
**SIGNIFICANCE**					
Solution (S)		^***^	^***^	ns	^***^
Graft combination (G)		^*^	^*^	ns	ns
S × G		^**^	ns	ns	ns

*E, cucumber hybrid “Ekron” (Cucumis sativus L.); E/F, “Ekron” grafted onto figleaf gourd rootstock (Cucurbita ficifolia Bouché); E/C, “Ekron” grafted onto squash hybrid rootstock “P360” (Cucurbita maxima Duch. × Cucurbita moschata Duch.)*.

*Values are the means of four replicate samples*.

*Means within columns separated using Duncan's multiple range test, P = 0.05*.

*ns*.,

*, **, *** *Nonsignificant or significant at P < 0.05, 0.01, or 0.001, respectively*.

### Mineral composition and partitioning

A different pattern of macronutrient accumulation and distribution in plant tissues was detected as a function of nutrient solution and grafting combination (Table 5). The concentration of N in leaf and fruit tissues were negatively affected by decreasing pH and the addition of 0.75 mM Al in the nutrient solution, while it remained unaffected in stems, fruits, and roots as compared with pH 6.0 (Table 5). Under Al stress conditions E/C plants markedly had higher N in their leaves than E/F and non-grafted plants (Table 5). Decreasing the pH from 6.0 to 3.5 in the nutrient solution and the further addition of Al significantly decreased P concentration in stems, fruits and roots (Table 5). The concentration of P in roots was lower in non-grafted than grafted plants in both treatments mentioned before (Table 5).

**Table 5:** **Effects of grafting combination, nutrient solution pH, and aluminum concentration on macronutrient composition of leaves, stems, fruits, and roots of cucumber plants grown in experiment 2**.

**Solution**	**Graft combination**	**Macronutrients (g kg^−1^ DW)**																			
		***N***				***P***				***K***				**Ca**				**Mg**			
		**Leaves**	**Stems**	**Fruits**	**Roots**	**Leaves**	**Stems**	**Fruits**	**Roots**	**Leaves**	**Stems**	**Fruits**	**Roots**	**Leaves**	**Stems**	**Fruits**	**Roots**	**Leaves**	**Stems**	**Fruits**	**Roots**
pH 6.0	E	44.9a	35.2	40.3	33.2	7.3	9.2	5.9	26.3a	33.2ab	40.5	36.0	10.8	42.4	10.1	3.7	6.3	7.4ab	4.9a	2.5	0.9
	E/F	43.6ab	28.7	37.5	32.9	5.7	8.6	6.8	25.9a	29.9b	35.9	35.3	13.8	42.6	8.0	3.5	6.3	7.0b	4.6ab	2.7	1.1
	E/C	45.2a	33.5	41.0	30.3	6.3	9.2	6.8	23.2a	39.4a	37.7	35.5	15.4	46.3	10.6	5.2	6.6	8.1a	5.3a	2.4	1.4
	Mean	44.6a	32.5	39.6a	32.1	6.5	9.0a	6.5a	25.1a	34.2a	38.0a	35.6a	13.3	43.8a	9.6	4.1a	6.4	7.5a	4.9a	2.6a	1.1
pH 3.5	E	42.5ab	30.3	35.8	27.0	6.4	9.1	6.1	10.8c	34.4ab	40.0	35.7	11.8	37.6	9.6	3.8	4.7	7.2ab	4.1b	2.4	0.8
	E/F	40.7b	28.5	32.0	28.9	5.1	8.7	5.8	17.1bc	29.2b	34.9	32.2	20.1	33.9	8.6	3.2	6.0	6.0b	4.2b	2.4	1.3
	E/C	42.1ab	29.3	35.7	30.0	5.6	9.4	6.0	20.0b	34.3ab	36.1	33.1	18.7	38.1	10.0	4.7	6.4	7.3ab	4.5b	2.2	1.5
	Mean	41.8b	29.4	34.5b	28.6	5.7	9.1a	6.0a	16.0b	32.6a	37.0a	33.6ab	16.9	36.5b	9.4	3.9a	5.7	6.8b	4.3b	2.3b	1.2
pH 3.5 + Al	E	33.0d	27.7	33.9	29.7	5.0	6.0	3.4	11.3c	32.5ab	39.4	31.2	15.5	26.0	10.6	2.8	5.5	4.9c	3.9b	2.1	1.2
	E/F	32.4d	25.8	32.0	31.1	5.7	6.9	4.2	16.5bc	27.8b	31.9	28.9	17.5	20.1	8.2	2.7	4.4	4.3c	3.8b	2.0	0.9
	E/C	37.9c	27.5	33.7	30.5	5.7	6.8	5.1	17.5b	29.1b	36.5	32.8	13.1	28.1	11.6	3.1	7.2	6.6b	4.1b	2.1	1.9
	Mean	34.4c	27.0	33.2b	30.4	5.4	6.5b	4.3b	15.1b	29.8b	35.9b	31.0b	15.3	24.7c	10.2	2.9b	5.7	5.3c	3.9c	2.0c	1.3
**SIGNIFICANCE**																					
Solution (S)		^***^	ns	^**^	ns	ns	^***^	^***^	^***^	^**^	^***^	^**^	ns	^***^	ns	^***^	ns	^***^	^**^	^***^	ns
Graft combination (G)		ns	ns	ns	ns	ns	ns	ns	^*^	^***^	ns	ns	ns	^*^	^***^	^***^	^*^	^***^	^*^	ns	^**^
S × G		^*^	ns	ns	ns	ns	ns	ns	^*^	^*^	ns	ns	ns	ns	ns	ns	ns	^**^	^**^	ns	ns

*E, cucumber hybrid “Ekron” (Cucumis sativus L.); E/F, “Ekron” grafted onto figleaf gourd rootstock (Cucurbita ficifolia Bouché); E/C, “Ekron” grafted onto squash hybrid rootstock “P360” (Cucurbita maxima Duch. × Cucurbita moschata Duch.)*.

*Values are the means of four replicate samples*.

*Means within columns separated using Duncan's multiple range test, P = 0.05*.

*ns*.,

*, **, *** *Nonsignificant or significant at P < 0.05. 0.01, or 0.001, respectively*.

The mean K concentration of the grafting combinations in stems and fruits decreased in both treatments (pH and pH + Al), more detrimental when Al was supplied compared with only pH 3.5 (Table 5). Comparing the grafting treatments, the lowest K concentration was found in E/F (Table 5). The Ca concentration was also significantly affected by the low pH in the nutrient solution and the addition of Al but compared with K not in the fruits but in the roots. At pH 3.5 + Al the leaves and fruits had significantly lower Ca concentration in comparison to those observed at pH 3.5 and control treatment (Table 5). Moreover, the concentration of Ca in all plant tissues (leaf, stem, fruit, and root) was higher in E/C than in E/F and in non-grafted plants.

Decreasing the pH and the addition of Al in the nutrient solution decreased significantly the Mg concentration in leaves, stems, and roots especially under Al stress. The concentration of Mg in leaves of Al-treated plants was significantly affected by the grafting combination with values recorded for E/C plants being higher than E/F and non-grafted plants. Similarly to leaves, the Mg concentration in roots was significantly higher in E/C than in other grafting combinations (Table 5).

The effect of low pH and Al-supply on tissue micronutrient concentrations was highly significant (Table 6). The concentration of Fe in leaf tissue decreased in both stressed plant treatments with the lowest values recorded in non-grafted plants supplied with pH 3.5 + Al. Concerning the grafting combination, the lowest concentration of Fe in the roots was observed in E/F plants (Table 6). Moreover, the concentrations of Mn (in all plant tissues), Zn (fruits and roots), and B (leaves) declined with increasing acidity and Al concentration in the nutrient solution. Finally, when averaged over nutrient solution, the Mn and Zn concentrations in all plant tissues were significantly higher in E/C as compared with other grafting combinations (Table 6).

**Table 6:** **Effects of grafting combination, nutrient solution pH and aluminum concentration on micronutrient composition of leaves, stems, fruits, and roots of cucumber plants grown in experiment 2**.

**Solution**	**Graft combination**	**Micronutrients (mg kg^−1^ DW)**															
		**Fe**				**Mn**				**Zn**				**B**			
		**Leaves**	**Stems**	**Fruits**	**Roots**	**Leaves**	**Stems**	**Fruits**	**Roots**	**Leaves**	**Stems**	**Fruits**	**Roots**	**Leaves**	**Stems**	**Fruits**	**Roots**
pH 6.0	E	56.5bc	30.6c	27.0	202.6	254.4	54.6ab	23.4c	64.2	48.0bc	40.9	32.5	28.0	67.6	15.8	13.2	7.8
	E/F	73.3ab	27.6c	30.6	123.5	269.8	41.1bc	23.7c	64.0	32.9d	49.1	30.0	77.4	82.5	16.3	13.9	10.5
	E/C	62.3b	43.9a	24.8	202.6	302.3	63.9a	35.6a	82.6	65.8a	61.5	30.8	56.8	68.1	14.7	13.3	10.7
	Mean	64.1	34.0ab	27.4a	176.2	262.1a	53.2a	27.6a	70.3a	48.9	50.5	31.1a	54.1b	72.7a	15.6	13.4	9.6
pH 3.5	E	59.8bc	35.9b	31.2	192.4	205.5	43.3bc	26.0bc	62.2	50.4b	53.3	30.3	29.9	58.3	14.8	12.7	11.7
	E/F	80.7a	39.0a	34.6	170.1	219.3	48.1b	22.2c	66.1	30.9d	49.7	27.6	63.4	57.9	16.3	13.4	13.7
	E/C	72.9ab	38.8a	29.2	176.7	248.5	48.1b	30.5b	71.4	55.5b	81.8	32.7	72.1	59.1	15.4	11.9	9.3
	Mean	71.1	37.9a	31.6a	179.7	212.4b	46.5b	26.2a	66.6a	45.6	61.6	30.2a	55.1b	58.4b	15.5	12.7	11.5
pH 3.5 + Al	E	48.0c	31.2bc	19.0	205.5	141.0	37.5b	15.9d	37.9	45.4c	55.2	23.3	64.3	52.3	16.9	13.1	7.7
	E/F	65.8ab	30.8c	19.6	130.2	132.4	42.6bc	17.4d	32.1	47.3c	62.5	22.2	86.8	61.9	18.2	13.6	10.4
	E/C	82.2a	30.3c	21.1	208.6	163.3	47.6b	18.7d	75.2	50.7bc	82.9	30.2	72.6	47.5	13.3	12.8	11.6
	Mean	65.3	30.8b	19.9b	181.4	136.7c	42.6b	17.4b	48.4b	47.8	66.9	25.2b	74.6a	53.9b	16.1	13.1	9.9
**SIGNIFICANCE**																	
Solution (S)		ns	^***^	^***^	ns	^***^	^***^	^***^	^*^	ns	ns	^***^	^*^	^***^	ns	ns	ns
Graft combination (G)		^***^	^*^	ns	^**^	^**^	^**^	^***^	^*^	^***^	^***^	^***^	^***^	ns	ns	ns	ns
S × G		^*^	^*^	ns	ns	ns	^*^	^*^	ns	^*^	ns	ns	ns	ns	ns	ns	ns

*E, cucumber hybrid “Ekron” (Cucumis sativus L.); E/F, “Ekron” grafted onto fig-leaf gourd rootstock (Cucurbita ficifolia Bouché); E/C, “Ekron” grafted onto squash hybrid rootstock “P360” (Cucurbita maxima Duch. × Cucurbita moschata Duch.)*.

*Values are the means of four replicate samples*.

*Means within columns separated using Duncan's multiple range test, P = 0.05*.

*ns*.,

*, **, *** *Nonsignificant or significant at P < 0.05. 0.01, or 0.001, respectively*.

### Leaf area, SPAD index, and electrolyte leakage

The total leaf area and electrolyte leakage were significantly affected by grafting combination, nutrient solution and their interaction, whereas the SPAD index was significantly influenced by grafting combination and nutrient solution with no interaction of these two factors (Table 7). The total leaf area decreased in response to a decrease of nutrient solution pH, with more detrimental at pH 3.5 + Al (Table 7). However, comparing pH 3.5 + Al with pH 6, the leaf area was less reduced in E/C (46%) than in E/F (61%) and non-grafted plants (68%; Table 7). The highest mean SPAD index of the grafting treatments was recorded at pH 6.0, while it was significantly lower at pH 3.5 + Al. The chlorophyll content recorded in grafted plants (avg. 40.8) was higher by 7% compared with ungrafted plants (38.3). Finally, the highest electrolyte leakage values were recorded in E/C and non-grafted plants supplied with pH 3.5 + Al (Table 7).

**Table 7:** **Effects of grafting combination, nutrient solution pH, and aluminum concentration on final leaf area, SPAD index, and leaf electrolyte leakage of cucumber plants grown in experiment 2**.

**Solution**	**Graft combination**	**Leaf area (m^2^ plant^−1^)**	**SPAD index**	**Electrolyte leakage (%)**
pH 6.0	E	1.24a	47.3	33.4e
	E/F	1.11a	49.1	35.0e
	E/C	1.05ab	48.3	38.6de
	Mean	1.13a	48.2a	35.7c
pH 3.5	E	0.73c	36.6	51.2c
	E/F	0.79bc	37.4	48.4d
	E/C	0.85b	41.0	42.8d
	Mean	0.79b	38.3ab	47.5b
pH 3.5 + Al	E	0.39e	31.1	68.3a
	E/F	0.43e	33.8	64.9a
	E/C	0.57d	35.2	61.1b
	Mean	0.46c	33.4b	64.8a
**SIGNIFICANCE**				
Solution (S)		^***^	^***^	^***^
Graft combination (G)		ns	^**^	ns
S × G		^*^	ns	^**^

*E, cucumber hybrid “Ekron” (Cucumis sativus L.); E/F, “Ekron” grafted onto figleaf gourd rootstock (Cucurbita ficifolia Bouché); E/C, “Ekron” grafted onto squash hybrid rootstock “P360” (Cucurbita maxima Duch. × Cucurbita moschata Duch.)*.

*Values are the means of four replicate samples*.

*Means within columns separated using Duncan's multiple range test, P = 0.05*.

*ns*.,

*, **, *** *Nonsignificant or significant at P < 0.05, 0.01, or 0.001, respectively*.

## Discussion

Under acidic soils, reduced plant growth and consequently productivity are induced by different morphological, biochemical, and physiological alterations (Kochian et al., 2015; Rengel et al., 2015). Rapid damage and growth inhibition of the root system is the primary symptom of Al toxicity at concentrations in the μM range, which may be associated with interference in the elongation and division of meristematic cells (Rengel and Zhang, 2003; Kochian et al., 2004; Sivaguru et al., 2013). This interference reduces the absorption of water and nutrients, thus conditioning stress sensitivity and limiting plant biomass production (Inostroza-Blancheteau et al., 2012). In agreement, in both experiments a significant reduction in root, shoot biomass, and yield was observed at low pH (3.5) and even more, when Al was added (Figure 1; Tables 1, 2), suggesting that these two stresses differ in their inhibition on plant growth (Rangel et al., 2005; Bose et al., 2010). Moreover, the reduction in plant biomass production depended on the Al concentration. The constrained crop growth and fruit yield observed under pH 3.5 and under pH 3.5 + Al has been reported in previous open field and greenhouse studies on tomato (Nogueirol et al., 2015) and zucchini squash (Rouphael et al., 2015). Nevertheless, decreasing pH and increasing Al concentration in the nutrient solution improved fruit quality characteristics such as fruit DM and TSS contents (Table 3). These phenomena have been found before for zucchini and explained by a decrease in water accumulation by the fruit without any effect on the synthesis and accumulation of organic solutes (Rouphael et al., 2012, 2015).

When cucumber cv. Ekron were grafted onto pumpkin rootstock “P360” compared with non-grafted and grafted onto figleaf gourd, the suppression of growth and production suppression under Al-supply was mitigated indicating a genetic diversity in Al-tolerance among cucumber rootstocks. The Al-tolerance of cucumber grafted onto pumpkin “P360” may be attributed to a greater uptake and translocation of K, Ca, Mg, Mn, and Zn to the aerial parts (leaves and stems). The enhanced nutritional status and abiotic stress-tolerance of grafted vegetables has often been associated with an enlarged and more vigorous root system (Colla et al., 2010c; Savvas et al., 2010). The root is the first organ sensing abiotic stresses in soil or substrates, such as acidity and Al toxicity. Therefore, the enhanced root growth (Table 1), length, and density (data not shown) of a pumpkin compared with a cucumber rootstock are important aspects for cucumbers cultivated in acidic conditions with or without Al toxicity.

Al accumulates in the epidermis and in the outer cortex of the root (Delhaize et al., 1993; Ryan et al., 2001). Many studies demonstrated that the major fraction of absorbed Al (30–90% of total Al) is localized in the apoplast (Yang et al., 2000; Ryan et al., 2001; Pereira et al., 2010). In Experiment 2, Al concentration in the root tissues was 50 times higher than in the cucumber leaves and even higher than in stems or fruits (Table 4). This indicates that the endodermis of roots represents a barrier to the transport of Al to the shoot (Dogan et al., 2014). Interestingly, this ratio was independent of the type of rootstock used although the absolute Al concentration differed between grafted and non-grafted root systems. The different Al accumulation in plant tissues highly depends on the mechanism (exclusion or internal tolerance) used by plants to confer tolerance to Al toxicity (Delhaize and Ryan, 1995; Kochian, 1995). Our results showed that Al concentration in cucumber roots was similar between grafted (E/C and E/F) and non-grafted plants grown under Al-stress (Table 4). This indicates that neither the figleaf gourd nor the pumpkin rootstocks were able to exclude Al from the root apex by exudation of organic acids (citrate, malate, and oxalate) into the rhizosphere (Brunner and Sperisen, 2013). On the other hand, the lowest Al accumulation in leaves with pH 3.5 + Al was recorded in the E/C combination compared to the other grafting combinations (Table 4) indicating that another mechanism was involved in mitigating the Al toxicity effect (Barceló and Poscheinrieder, 2002; Brunner and Sperisen, 2013). It appears that grafting cucumber onto pumpkin rootstock can restrict the Al root-to-shoot translocation (Table 4), throughout “the sequestration of this toxic element into less sensitive parts of the plant and cell compartments (e.g., vacuoles)” (Inostroza-Blancheteau et al., 2012; Brunner and Sperisen, 2013; Kochian et al., 2015). This suppression of Al uptake and translocation to shoots of grafted plants is consistent with previous reports indicating that appropriate rootstocks of *cucurbitaceous* and *solanaceous* species may limit the uptake of toxic elements and heavy metals (Edelstein et al., 2005; Rouphael et al., 2008a; Kumar et al., 2015b). Authors show that grafting annual fruit crops, such as melon, cucumber, and tomato onto vigorous rootstocks (i.e., pumpkin for cucurbits and “Maxifort” for tomato) may significantly restrict trace elements' (B and Cu) and heavy metal (Ni) concentrations in leaf and fruit tissue, thereby alleviating their detrimental effects on crop productivity and human health *via* the food chain (Savvas et al., 2010).

Nutrient uptake is a crucial aspect for the maintenance of homeostasis and plant growth under unfavorable soil conditions (i.e., edaphic stress; Seguel et al., 2013). Tolerance of Al is mostly reflected in limited alterations of macro and micronutrient's acquisition and translocation (Andrade et al., 2009). In the present study, decreasing pH level and increasing Al concentration in the nutrient solution depressed concentrations of macro- (N, P, K, Ca, and Mg) and micro-elements (Mn and Zn) in shoots (leaves and/or stems) and to a lesser degree in roots in both grafted and non-grafted plants (Tables 5, 6). Therefore, excessive Al accumulation affected uptake, translocation and accumulation of these nutrients in plant tissues, and hence was responsible for mineral imbalances and deficiencies as well as for the depression of plant growth and yield (Rouphael et al., 2015). Studies have demonstrated that Al interferes directly with several different plasma membrane channel proteins, thus reducing the uptake of mono- and divalent cations such as K and Ca (Gassmann and Schroeder, 1994; Piñeros and Tester, 1995). Al-stress has been reported to reduce Ca through three mechanisms: “(1) inhibition of Ca transport via symplasm by Al, (2) disruption of Ca homeostasis in cytoplasm by Al, and (3) Ca displacement by Al from apoplasm” (Delhaize and Ryan, 1995; Kochian, 1995). In addition to the reduction in K and Ca uptake, Al can also decrease Mg uptake in aerial parts (Rengel and Robinson, 1989), as we can confirm with our current data. The competition between these two cations (Al and Mg) was demonstrated for membrane transporters and metal binding sites in enzymatic reactions (Rengel and Robinson, 1989; Pécsváradi et al., 2009).

However, grafted and non-grafted plants diverged in their tissue nutrient concentration in relation to acidity and Al toxicity. Grafting cucumber plants onto the pumpkin rootstock (E/C) increased K, Ca, Mg, Mn, and Zn in shoot and/or root (Tables 5, 6) suggesting that selecting appropriate rootstocks can maintain nutrient homeostasis under Al-stress. The lower reduction in cation uptake observed in the E/C combination under Al-stress, could be associated to the lower inhibition of root growth and elongation since cation (especially K) accumulation contributes to the expansion of cell elongation (Cristancho et al., 2011). Similarly, the shoot and root Mg concentrations were higher in E/C compared to the other grafting combinations. It remains to be elucidated whether higher Mg uptake in E/C combination is a consequence of a greater root growth and/or greater Al tolerance of Mg-uptake systems (Rengel et al., 2015). From above we could suggest that an efficient metabolism system exists in E/C combination under Al toxicity, indicating that Al tolerance is correlated with enhanced mineral nutrient concentrations in plant tissues (Rouphael et al., 2015). These results are consistent with several studies (Rouphael et al., 2008a; Kumar et al., 2015a,b) reporting that rootstocks used in grafting of annual fruit crops were able to enhance the uptake of some macro- and micro-elements under heavy metal (e.g., Cd, Ni, and Cu) toxicity.

High Al concentrations in soil or substrates hamper plant development at a physiological and biochemical level, affect its photosynthetic rate, total chlorophyll content, and also inhibit electron transport in PSII (Chen, 2006; Inostroza-Blancheteau et al., 2012). This was confirmed with our measurements of the SPAD index, widely used as a non-destructive estimate of chlorophyll content. It dropped sharply in leaves of Al stressed non-grafted plants compared to both E/C and E/F plants (Table 7). This suggests the occurrence of chlorophyll degradation and early senescence, likely due to the harmful effects of reactive oxygen species on chloroplasts (Chen, 2006; Rouphael et al., 2015). Contrarily, the grafted cucumber plants in particular E/C combination were able to maintain a higher chlorophyll content in both stress treatments, thus exhibiting the highest yield and biomass. In addition to a reduced chlorophyll content, reductions in total leaf area at low pH and especially at Al-supply could be caused by a premature leaf senescence and ion toxicity (Seguel et al., 2013). Maintenance of a large leaf area upon Al stress in E/C combination (Table 7) may be crucial to guarantee production and translocation of photosynthates to the fruits, thus increasing the final yield in cucumber.

Adverse pH conditions and Al-stress induced impairment of membrane integrity and affect all physiological activities linked to membrane functioning (Rouphael et al., 2015). This is related to the fact that Al-enhanced oxidative stress caused by an increased production of reactive oxygen species leads to lipid and protein oxidation (Boscolo et al., 2003; Jones et al., 2006). The results of Experiment 2 indicate that in cucumber the electrolyte leakage percentage (i.e., degree of cell membrane injury) was significantly increased, and depended on the nutrient solution pH and the Al concentration (Table 7). The degree of cell membrane injury induced by Al-stress has often been related to calcium concentration, an important factor in increasing structural stability of cell membrane (Borer et al., 2005). Increasing Al concentration in the nutrient solution reduced leaf and root calcium uptake resulting in a reduction of cell membrane integrity (Pereira et al., 2010). However, E/C plants reduced the amount of ion leakage in Al-stress treatment by improving the Ca uptake in shoot and root tissues indicating that grafting cucumber onto pumpkin rootstocks has facilitated the membrane functions (i.e., semipermeability).

## Conclusions

Our findings indicate that in both grafted and non-grafted plants, agronomical, physiological, and mineral composition responses were negatively affected by acidity and Al concentration, with Al-stress being more phytotoxic than low pH treatment. The results of both experiments were able to verify our hypothesis that grafting onto suitable rootstocks (i.e., pumpkin) may limit the Al accumulation in the aerial parts, improve the uptake of nutrients (K, Ca, Mg, Mn, and Zn), enhance chlorophyll synthesis as well as the root genotypes to control the cell membrane stability; thus alleviating the impacts of adverse pH level and Al toxicity on crop productivity. These results might be useful to assist the selection of tolerant rootstocks in breeding programs particularly in acidic soils, where Al-toxicity is a major agronomic constraint.

## Author contributions

YR performed the long term experiment with agronomical and physiological analysis and he was involved in writing the manuscript (Experiment 2); ER and MC performed the mineral analysis in both experiments and they collaborated in manuscript preparation; MB and DS were involved in the short term experiment (Experiment 1) and in manuscript preparation; GC defined the scientific hypothesis, set up the experimental protocols, made the statistical analysis of experimental data, and he was involved in the manuscript preparation.

### Conflict of interest statement

The authors declare that the research was conducted in the absence of any commercial or financial relationships that could be construed as a potential conflict of interest.
